# Blood–brain barrier permeable nano immunoconjugates induce local immune responses for glioma therapy

**DOI:** 10.1038/s41467-019-11719-3

**Published:** 2019-08-28

**Authors:** Anna Galstyan, Janet L. Markman, Ekaterina S. Shatalova, Antonella Chiechi, Alan J. Korman, Rameshwar Patil, Dmytro Klymyshyn, Warren G. Tourtellotte, Liron L. Israel, Oliver Braubach, Vladimir A. Ljubimov, Leila A. Mashouf, Arshia Ramesh, Zachary B. Grodzinski, Manuel L. Penichet, Keith L. Black, Eggehard Holler, Tao Sun, Hui Ding, Alexander V. Ljubimov, Julia Y. Ljubimova

**Affiliations:** 1 Nanomedicine Research Center, Department of Neurosurgery, Cedars-Sinai Medical Center, 8700 Beverly Blvd, AHSP, Los Angeles, CA 90048 USA; 2 Bristol-Myers Squibb, 700 Bay Road, Redwood City, CA 94063 USA; 3 Department of Pathology and Laboratory Medicine, Cedars-Sinai Medical Center, 8700 Beverly Blvd., ST 8719, West Hollywood, CA 90048 USA; 4 Department of Biomedical Sciences, Board of Governors Regenerative Medicine Institute, Cedars-Sinai Medical Center, 8700 Beverly Blvd, AHSP, Los Angeles, CA 90048 USA; 5 Samuel Oschin Comprehensive Cancer Center, Cedars-Sinai Medical Center, 8700 Beverly Blvd, Los Angeles, CA 90048 USA; 6 Harvard Medical School, 25 Shattuck Street, Boston, MA 02115 USA; 7 University of California, Los Angeles (UCLA), 621 Charles E Young Dr S, Los Angeles, CA 90095 USA; 8 Division of Surgical Oncology, Department of Surgery, David Geffen School of Medicine at University of California, Los Angeles (UCLA), 10833 Le Conte Ave, Los Angeles, CA 90095 USA; 9 Department of Microbiology, Immunology and Molecular Genetics, David Geffen School of Medicine at University of California, Los Angeles (UCLA), Los Angeles, CA USA; 10 Jonsson Comprehensive Cancer Center, University of California, Los Angeles (UCLA), 10833 Le Conte Ave, Los Angeles, CA 90095 USA; 11 The Molecular Biology Institute, University of California, Los Angeles (UCLA), 611 Charles E Young Dr E, Los Angeles, CA 90095 USA; 12 AIDS Institute, University of California, Los Angeles (UCLA), 10940 Wilshire Blvd Suite 960, Los Angeles, CA 90024 USA; 13 The California NanoSystems Institute, University of California, Los Angeles (UCLA), 570 Westwood Plaza Building 114, Los Angeles, CA 90095 USA; 14 Institut für Biophysik und Physikalische Biochemie, Universität Regensburg, Regensburg, D-93040 Germany

**Keywords:** Cancer microenvironment, CNS cancer

## Abstract

Brain glioma treatment with checkpoint inhibitor antibodies to cytotoxic T-lymphocyte-associated antigen 4 (a-CTLA-4) and programmed cell death-1 (a-PD-1) was largely unsuccessful due to their inability to cross blood–brain barrier (BBB). Here we describe targeted nanoscale immunoconjugates (NICs) on natural biopolymer scaffold, poly(β-L-malic acid), with covalently attached a-CTLA-4 or a-PD-1 for systemic delivery across the BBB and activation of local brain anti-tumor immune response. NIC treatment of mice bearing intracranial GL261 glioblastoma (GBM) results in an increase of CD8+ T cells, NK cells and macrophages with a decrease of regulatory T cells (Tregs) in the brain tumor area. Survival of GBM-bearing mice treated with NIC combination is significantly longer compared to animals treated with single checkpoint inhibitor-bearing NICs or free a-CTLA-4 and a-PD-1. Our study demonstrates trans-BBB delivery of tumor-targeted polymer-conjugated checkpoint inhibitors as an effective GBM treatment via activation of both systemic and local privileged brain tumor immune response.

## Abstract (web summary)

Glioma therapy with checkpoint inhibitors has limited blood–brain barrier (BBB) penetration and therapeutic effects. Here, the authors develop nanopolymer-conjugated checkpoint inhibitors and show their trans-BBB delivery and anti-glioma efficacy.

## Introduction

Glioblastoma multiforme (GBM) is the most common and most aggressive form of primary brain tumor in adults, with an incidence of 3.2 per 100,000 population. Comprehensive advances in molecular profiling including our work under The Cancer Genome Atlas (TCGA) project for GBM consortium have led to the identification of key prognostic factors^1,2^, but this has not translated into change in therapy or survival^3,4^. Immunotherapy is one of the fastest developing approaches in clinical oncology with successful treatment of different cancers^5^. However, the unique immune environment of the central nervous system (CNS) needs consideration when pursuing immunotherapeutic approaches for gliomas. Treatment options are limited, in part because of inefficient drug delivery across the blood–brain barrier (BBB)^6–8^. A recently published review^9^ summarized the results of immunotherapy clinical trials in glioma: 28 clinical trials for vaccines (e.g., a peptide vaccine that targets EGFRvIII or IDH1); 13 clinical trials completed for oncolytic viruses; 15 phase III clinical trials for checkpoint inhibitor antibodies (e.g., CheckMate 143 trial); and genetically modified T cells expressing chimeric antigen receptors (CAR-T cells). Unfortunately, no treatment so far has been superior to the standard-of-care for GBM, represented by temozolomide/radiation therapy with 14.6 months average survival^9^.

Blockade of cytotoxic T-lymphocyte-associated antigen 4 (CTLA-4) using the antagonistic monoclonal antibody (mAb) ipilimumab was the first strategy to achieve a significant clinical benefit for stage IV melanoma patients^10,11^. Humanized mAbs against immune system response modulators CTLA-4 (ipilimumab), and programmed cell death-1 (PD-1) (pembrolizumab and nivolumab), received FDA approval. Their effect is due to the suppression of regulatory T cells (Tregs) and activation of anti-tumor immune response by cytotoxic T lymphocytes (CTLs). Systemic administration of CTLA-4 or PD-1 and programmed cell death ligand 1 (PD-L1) mAbs can suppress some tumors, but has low efficacy against brain tumors^9,12,13^.

Despite growing evidence to support an interaction between the CNS and general immune system^14^, clinical trials using nivolumab and ipilimumab in GBM showed serious safety issues, but not a significant anti-tumor treatment effect^15^. Although CTLA-4, PD-1 and other antibodies do not cross the BBB^16–18^, some efficacy has been demonstrated against GBM, possibly due to general immune system activation.

Recent studies highlighted significant roles of the tumor microenvironment in tumor development and progression. Tumor-associated macrophages/microglia (TAMs) are a major stromal cell component in GBM. It was shown that both mouse and human TAMs express PD-1. TAM PD-1 expression increases over time in mouse cancer models and with higher disease stage in human cancers. PD-1 expression by TAMs inhibits phagocytosis and tumor immunity^19–21^. Macrophage polarization into M1 and M2 phenotypes with distinct functional consequences is well established, and M1 anti-tumor macrophages have shown promise to function as a cancer immunotherapy^22,23^. M1 macrophages, in response to IFNγ or TNFα, convert arginine into nitric oxide (NO) through inducible nitric oxide synthase (iNOS) to promote anti-tumor activity. M2 macrophages, polarized by TGFβ and IL-10, accumulate in tumors and can induce Treg cells that suppress CTLs. They can also inhibit activation of NK cells through immunosuppressive TGFβ. The immune response to tumors appears to be the result of interactions between T cells (Tregs and effector cells), NK cells, and TAMs^22,23^.

In order to achieve successful immunotherapeutic effects in gliomas, the corresponding drugs should be able to cross the BBB and reach the tumor. We combine nanotechnology and immunotherapy advances^23,24^ to deliver nanoscale immunoconjugate (NIC) drugs across the BBB and treat GBM. A versatile drug carrier, poly(β-l-malic acid) (PMLA), a natural polymer obtained from the slime mold *Physarum polycephalum*^25,26^, is used to deliver covalently conjugated CTLA-4 and PD-1 antibodies (a-CTLA-4 and a-PD-1) to brain tumor cells, which results in local immune system activation and prolonged survival of intracranial GBM GL261-bearing mice. PMLA-based nanotherapeutics target brain tumors by crossing the BBB using transferrin receptor (TfR)-mediated transcytosis^25^. We also use an alternative mechanism of delivery through BBB with PMLA-conjugated Angiopep-2 (AP-2) peptide, which is a synthetic low-density lipoprotein receptor-related protein 1 (LRP-1) ligand^27^. To our knowledge, this is the first successful use of polymer-based carriers with covalently attached immunotherapeutics to activate local immune response and treat brain tumors.

## Results

### NIC synthesis

**Fig. 1:**
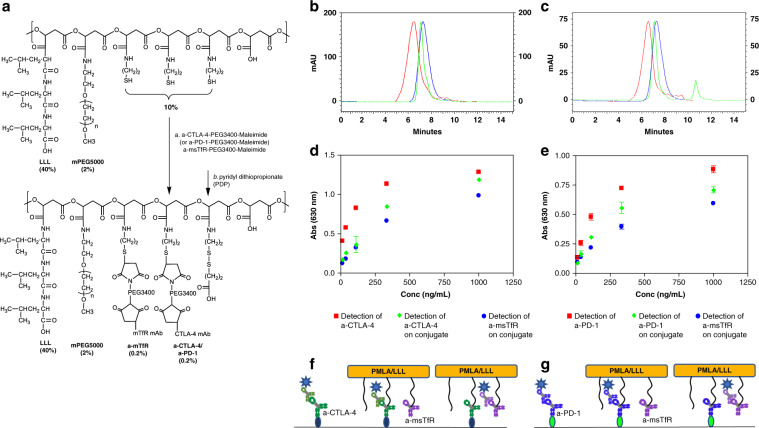
Synthesis and characterization of NICs. **a** Synthesis of P/mPEG5000(2%)/LLL(40%)/a-msTfR(0.2%)/a-CTLA-4 (0.2%). The upper structure represents the pre-conjugate and the lower structure represents the final nanoconjugate with antibodies and 3-(2-pyridyldithio) propionate (PDP). In some experiments, a-msTfR was substituted by AP-2 peptide (TFFYGGSRGKRNNFKTEEY). **b** SE-HPLC analysis of P/mPEG5000(2%)/LLL(40%)/a-msTfR(0.2%)/a-CTLA-4(0.2%). Right blue peak, the pre-conjugate P/mPEG5000(2%)/LLL(40%)/MEA(2%); middle (green) peak, a-CTLA-4; red peak, the synthesized P/mPEG5000(2%)/LLL(40%)/a-msTfR(0.2%)/a-CTLA-4(0.2%). **c** SE-HPLC analysis of P/mPEG5000(2%)/LLL(40%)/a-msTfR(0.2%)/a-PD-1(0.2%). Right blue peak, the pre-conjugate P/mPEG5000(2%)/LLL(40%)/MEA(2%); middle (green) peak, a-PD-1; red peak, the synthesized P/mPEG5000(2%)/LLL(40%)/a-msTfR(0.2%)/a-PD-1(0.2%). **d** and **e** Validation of simultaneous conjugation and activity of a-msTfR and a-CTLA-4/a-PD-1 on a single platform by ELISA of P/a-CTLA-4 **d** and P/a-PD-1 **e**. a-CTLA-4 and a-PD-1 (■), a-CTLA-4 and a-PD-1 on conjugate (◆), a-msTfR (●). **f** Illustration of ELISA method used in **d**. **g** Illustration of ELISA method used in **e**

We covalently attached checkpoint inhibitor antibodies, a-CTLA-4 IgG2b or a-PD-1 IgG, to the PMLA backbone to reach stability for plasma circulation. mPEG5000 was attached for solubility and stability, anti-mouse TfR antibody (a-msTfR), to cross the BBB, and trileucine (LLL), for stabilization of PMLA against hydrolytic degradation^28^, hydrophobization, and for endosomolytic drug delivery^29^. Synthesis has been adapted from previous studies^25,26,28,29^ with the analysis specific to our NICs (Fig. 1, see Supplementary Methods). The pre-conjugate was analyzed by physico-chemical methods. The bound free −SH group was determined to be 7% using Ellman assay. The content of LLL and mPEG was determined to be 44% and 2.5% by 1H NMR^6^ and by colorimetric assay using ammonium ferrothiocyanate^30^, respectively. Synthesis of NICs for polymer-conjugated a-CTLA-4, as an example, involved synthesis of pre-conjugate P/mPEG5000(2%)/LLL(40%)/MEA(10%), chemical activation of mAb maleimide, a-CTLA-4-PEG3400-maleimide, and a-msTfR-PEG3400-maleimide, and conjugation of pre-conjugate with activated mAbs through thiol ether formation (Fig. 1a). This was followed by blocking residualfree thiol groups. Size-exclusion HPLC (SE-HPLC) confirmed conjugation (Fig. 1b, c). Fourier transform infrared spectroscopy (FTIR) and pull-down ELISA further validated the structure of P/mPEG5000(2%)/LLL(40%)/a-msTfR(0.2%)/a-CTLA-4(0.2%) (P/a-CTLA-4) (Fig. 1d, f; Supplementary Fig. 1A, C) and P/mPEG5000(2%)/LLL(40%)/a-msTfR(0.2%)/a-PD-1(0.2%) (P/a-PD-1) (Fig. 1e, g; Supplementary Fig. 1B, D). Total amount of malic acid for treatment was analyzed by malate dehydrogenase assay (Supplementary Methods). The amount of total mAb of each NIC was quantitated with Pierce Protein BCA assay kit (Thermo Fisher Scientific) using a-CTLA-4 as standard. The loading of a-CTLA-4 and a-PD-1 in NICs was 0.21% in P/a-CTLA-4, 0.26% in P/a-PD-1, and 0.24% in NIC combination group determined by dividing total mole of antibody by total mole of malic acid per injection (Supplementary Table 1).

Free a-CTLA-4, a-PD-1, and a-msTfR showed somewhat higher binding affinity toward their respective antigens on a plate surface compared with polymer-bound antibodies, due to the bulkier size of the NICs. In addition, we proved the presence of pairs of two antibodies (i.e. a-CTLA-4 and a-msTfR or a-PD-1 and a-msTfR) within one single polymer chain using pull-down ELISA (Fig. 1f, g, Supplementary Fig. 1C, D). The ELISA signal intensity of a-msTfR on P/a-CTLA-4 conjugate was comparable to that of a-CTLA-4 on the same conjugate, confirming the presence of both a-msTfR and a-CTLA-4 on the conjugate (Fig. 1d). Similar results were obtained for P/a-PD-1, when the surface was coated with PD-1 (Fig. 1e), and for P/a-CTLA-4 and P/a-PD-1, when the surface was coated with msTfR (Supplementary Fig. 1A, B). ELISA results confirmed the reactivity and the presence of each antibody in the conjugates. FTIR analysis for P/a-CTLA-4 shows peaks at the O-H stretching frequencies of 2880 cm^−1^ (carboxylic acid O–H) that can be seen in both pre- and final NIC, as well as peaks that are present at the a-CTLA-4 spectrum, which are also present in the final NIC (mainly peaks at 3270 and 2953 cm^−1^, which are N–H and O–H stretching frequencies, and lower frequencies peaks at 1120 and 1630 cm^−1^) (Supplementary Fig. 2A). A similar trend could also be seen for P/a-PD-1, with significant specific peaks at 2875, 1664, 1031, and 942 cm^−1^ (Supplementary Fig. 2B). The analysis of the FTIR spectra, together with SE-HPLC and ELISA results, suggested that the antibodies were conjugated successfully to the pre-conjugate.

ζ-Potentials of NICs were in the range of −9.9 to −11.0 mV, reflecting the design and the intrinsic antibody charges. Hydrodynamic size was in the range of 28.0–28.5 nm by intensity, in agreement with design and molecular masses of constituents and the absence of aggregates, and endotoxin level was reduced below 0.1 EU/mL by phase separation method^31^ (Supplementary Table 1).

### NICs cross the BBB and reach the tumor interstitium

**Fig. 2:**
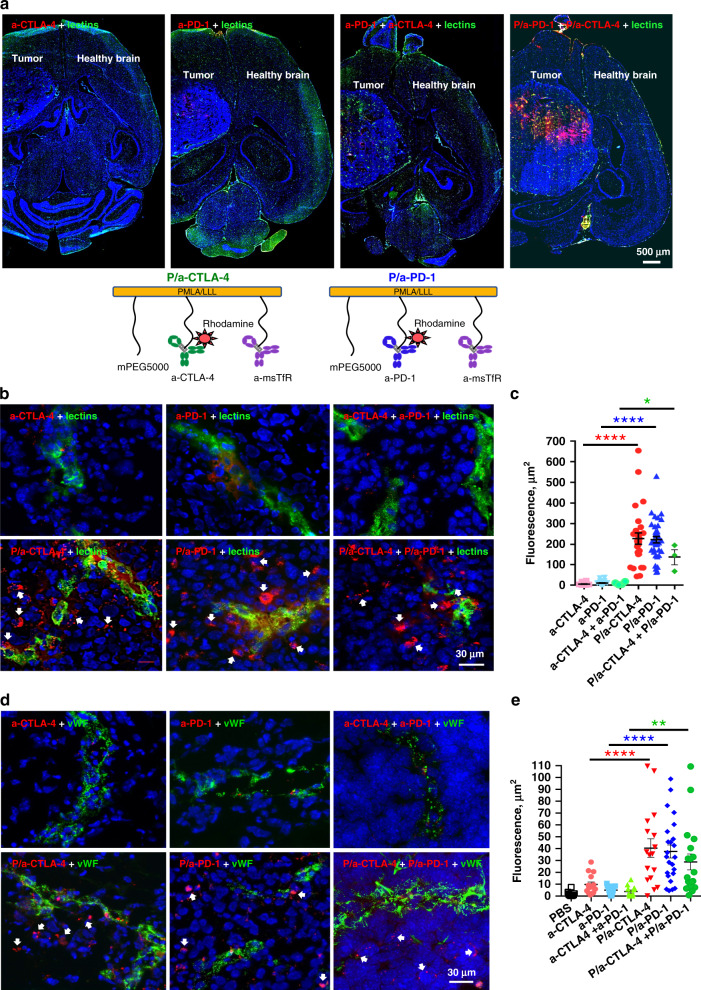
BBB crossing and glioma accumulation of fluorescently labeled NICs. **a** Top: drug distribution on whole brain sections after I.V. administration. Combined rhodamine-labeled NICs (a-CTLA-4+a-PD-1) show significant BBB crossing and tumor accumulation (red, right panel). Drug distribution heterogeneity may be related to regional variations in tumor vascularity. Free rhodamine-labeled mAbs (a-CTLA-4 or a-PD-1 or their combination) have little tumor accumulation, mostly inside the blood vessels stained with tomato and RCA120 lectins (green; left and middle). Healthy brain shows no drug accumulation. Part of the tumor was taken for other analyses. Representative pictures are shown. Scale bar applies to all images. Bottom: structure of NICs: left, PMLA/LLL/mPEG5000(2%)/PEG3400-a-msTfR (0.2%)/PEG3400-a-CTLA-4-rhodamine (0.2%); right, PMLA/LLL/mPEG5000(2%)/PEG3400-a-msTfR (0.2%)/PEG3400-a-PD-1-rhodamine (0.2%). **b** Drug distribution on brain tumor sections at high magnification. Blood vessels are stained with lectins (green). Free rhodamine-labeled (red) mAbs (a-CTLA-4 or a-PD-1 or their combination) are virtually absent outside of the blood vessels (top row). All NICs (red, arrows) are distributed mostly outside the blood vessels in the tumor parenchyma (bottom row). **c** Quantitative analysis of drug distribution in the tumor parenchyma. NIC treatments result in significantly more drug in the tumor parenchyma than treatments with free antibodies. Three to seven images of different brain sections per animal are used. Red fluorescence outside the vessels was quantitated as positive area in µm^2^ ± SEM. *p < 0.05; ****p < 0.0001 (one-way ANOVA with Sidak’s posttest). **d** Drug distribution on brain tumor sections at high magnification. Blood vessels are immunostained for von Willebrand factor (vWF, green). Free rhodamine-labeled (red) mAbs (a-CTLA-4 or a-PD-1 or their combination) are only seen inside the brain blood vessels (top row). All NICs (red, arrows) are distributed mostly in the tumor parenchyma (bottom row). **e** Quantitative analysis of drug distribution in the tumor parenchyma. NIC treatments result in significantly more drug outside of the blood vessels than free antibodies treatments. Five to seven images of six different brain sections per animal are used. Red fluorescence outside the vessels is quantitated as positive area in µm² ± SEM. **p < 0.01; ****p < 0.0001 (one-way ANOVA with Sidak’s posttest)

Rhodamine-labeled a-CTLA-4 and a-PD-1 or their combination could hardly be detected outside of the blood vessels positive for lectins (tomato and RCA120) in the intracranial tumors at 4 and 6 h after intravenous (I.V.) injection, indicating their inability to cross the BBB and reach the tumor parenchyma (Fig. 2a top; 2b top row). In contrast, rhodamine-labeled checkpoint inhibitor antibodies covalently attached to the polymer, P/a-CTLA-4 and P/a-PD-1 (schematics on Fig. 2a bottom) or their combination, were readily detected in the tumor parenchyma 4 h after I.V. injection (Fig. 2a, b bottom row). NICs were distributed through the tumor area (but not in the healthy brain), mostly outside the blood vessels, with only occasional presence in the vessels (Fig. 2a, b). Very similar results were obtained after vessel labeling for von Willebrand factor (Fig. 2c). Thus, NICs cross the BBB, allowing conjugated mAbs to bind to CTLA-4 and PD-1 and modulate the immune response in the tumor area. Optical imaging data analysis performed with ImageJ Fiji^6,32^ revealed significant differences (*F* = 46.52, DF = 5, *p* < 0.0001 for lectins; and *F* = 11.36, DF = 6, *p* < 0.0001 for von Willebrand factor by ANOVA) between fluorescent areas outside the vessels for NICs vs. free mAbs (Fig. 2c, e). Multiple pairwise comparisons by ANOVA with Sidak’s posttest also showed significantly more labeled NIC-attached checkpoint inhibitors or their combination than the free ones or their combination in the tumor parenchyma using both vessel labeling methods (Fig. 2c, e).

### NICs stimulate T cell and macrophage response in tumors

**Fig. 3:**
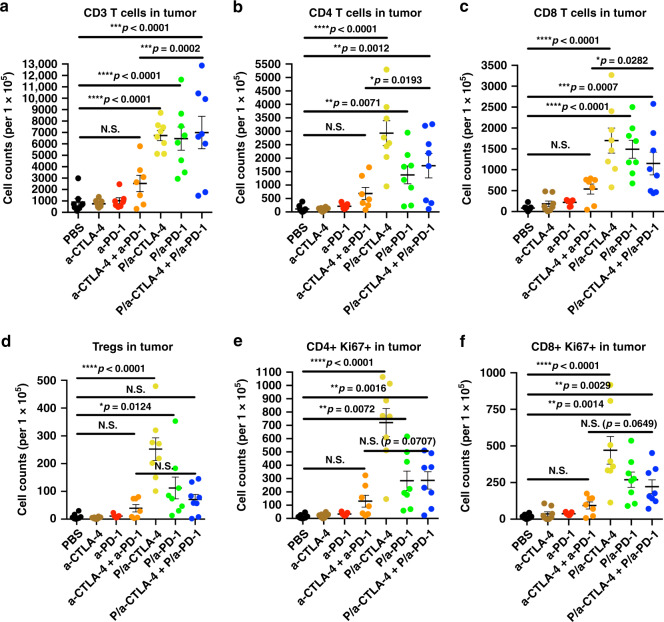
Increase of tumor infiltrating T cells revealed by flow cytometry. GL261 brain tumors were analyzed by the spectral flow cytometry (SONY Biotechnology) and tumor-associated T cells are presented as the cell counts in each treatment group. **a** CD3+ T cells; **b** CD3+CD4+ T helpers; **c** CD3+CD8+ T effectors; **d** CD3+CD4+Foxp3+ Tregs; **e** Ki67+ proliferating CD4+ cells; **f** Ki67+ proliferating CD8+ cells. Approximately 100,000 events/sample were recorded and analyzed by the SA3800 software (SONY Biotechnology). Data are mean ± SEM. *p < 0.05;. **p < 0.01; ***p < 0.001; ****p < 0.0001; N.S. = non-significant (one-way ANOVA with Sidak’s posttest). *N* = 6 per group

Independent in vivo experiment was conducted to treat mice with intracranially inoculated 2 × 10^4^ GL261 cells. Seven groups of mice (*n* = 8/group) were treated five times with free mAbs, their combination or NICs alone or in combination. Flow cytometry analysis (gating strategy presented on Supplementary Figs. 3 and 4) showed a significant increase of the CD3+, CD4+ and CD8+ T cell populations (*F* = 15.15, 12.91, 13.06, respectively, DF = 6, by ANOVA with Sidak’s posttest, *p* < 0.0001) in the tumor tissue after treatment with NICs, especially in animals treated with P/a-CTLA-4, P/a-PD-1, and their combination (co-injection), compared to PBS and free mAbs or their combination (Fig. 3a–c). The Treg fraction (CD4+FoxP3+) significantly increased in individual NIC groups only vs. PBS in pairwise comparisons (*F* = 14.19, DF = 6, by ANOVA with Sidak’s posttest, *p* < 0.0001). NIC combination did not significantly increase Tregs vs. either PBS or free mAbs or their combination (Fig. 3d). Tregs maintain tolerance to self-antigens and have a tendency to accumulate in tumors. Even after treatments, their numbers did not decrease, which agrees with recent data showing lack of Treg depletion by checkpoint inhibitor antibodies^5^. Proliferating CD4+Ki67 fraction was significantly growing in all NIC treatment groups vs. PBS (*F* = 19.08, DF = 6, by ANOVA with Sidak’s posttest, *p* < 0.0001; Fig. 3e) but the difference from free antibody combination did not reach significance. Importantly, CD8+ and especially proliferating CD8+Ki67+T cell fraction representing CTL was significantly increased in all NICs vs. PBS (*F* = 12.77, DF = 6, by ANOVA with Sidak’s posttest, *p* < 0.0001; Fig. 3f). This increase attested to the efficacy of treatment meant to activate CTLs by modulating Treg function.

**Fig. 4:**
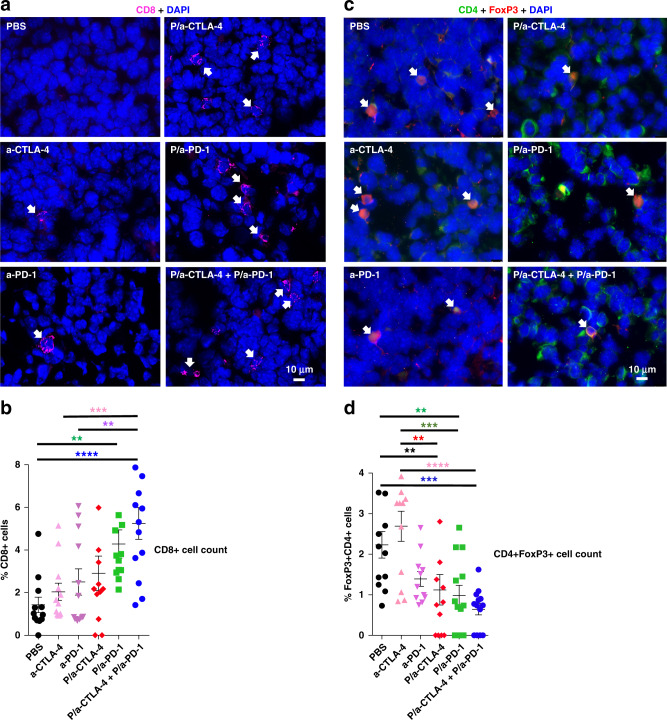
CD8+ increase and CD4+FoxP3+ decrease in glioma after NIC treatment. **a** Immunostaining of CD8+ T cells in tumors treated with PBS, a-CTLA-4, a-PD-1, P/a-CTLA-4, P/a-PD-1, and P/a-CTLA-4+P/a-PD-1. Arrows indicate CD8+ lymphocytes (magenta) that increase after NIC treatment. Scale bar on bottom right panel applies to all other images. **b** Treatments with P/a-PD-1 and P/a-CTLA-4+P/a-PD-1 result in a statistically significant increase of CD8+ lymphocytes inside the tumor. 8499 cells are analyzed in tumors from 24 mice representing six treatment groups, *N* = 4 per group. Data are mean ± SEM. **p < 0.01; ***p < 0.001; ****p < 0.0001 (one-way ANOVA with Sidak’s posttest). **c** Immunostaining of CD4+FoxP3+ T cells in tumors treated with PBS, a-CTLA-4, a-PD-1, P/a-CTLA-4, P/a-PD-1, and P/a-CTLA-4+P/a-PD-1. Arrows indicate CD4+FoxP3+ lymphocytes that are decreased after NIC treatment. CD4 staining is green, FoxP3 staining is red, DAPI nuclear staining is blue. **d** P/a-PD-1 and P/a-CTLA-4+P/a-PD-1 treatments significantly decrease the number of CD4+FoxP3+ lymphocytes in the tumor. 9898 cells are analyzed in tumors from 24 mice representing six treatment groups, *N* = 4 per group. **p < 0.01; ***p < 0.001; ****p < 0.0001 (one-way ANOVA with Sidak’s posttest)

To further study the local immune response, ex vivo brain tumors were collected near the end point of mice after treatment with NICs. CD8+ and CD4+FoxP3+ T cells were revealed by immunofluorescent staining of brain tumor cryosections and quantitated using Fiji software. For CD8+ staining, we analyzed 8499 cells in tumors from 24 mice representing different treatment groups, four mice per group (Fig. 4a, b). On average, each analyzed image contained 116 ± 25 cells, and cell densities were uniform for all data included in our analysis. We observed a significant increase in the number of CD8+ T cells in tumor tissue after NIC treatment (*F* = 5.383, DF = 5, by ANOVA with Sidak’s posttest; *p* = 0.0003; Fig. 4b). The percentage of CD8+ T cells was significantly increased vs. PBS group following treatments with P/a-PD-1 (*p* < 0.007) and P/a-CTLA-4+P/a-PD-1 (*p* < 0.0001). CD8+ T cell percentage was also significantly increased after P/a-CTLA-4+P/a-PD-1 compared to treatment with a-CTLA-4 (*p* = 0.001) and a-PD-1 (*p* < 0.006) (Fig. 4b). The NIC combination showed a tendency for increase in CD8+ T cells vs. NICs with single antibodies but it did not reach significance. Overall, the combined NIC treatment was the most effective at recruiting CD8+ T cells into the tumor tissue.

To determine the effect of our drug treatments on Treg in tumors, we also measured the percentage of CD4 and FoxP3 double immunolabeled T cells. Cells that were exclusively FoxP3+ were not counted. We analyzed 9898 cells in tumor tissues from 24 mice (four mice per group) (Fig. 4c, d). On average, each analyzed image contained 127 ± 26 cells; cell densities were uniform. We observed a significant decrease in the incidence of CD4+FoxP3+T cells in treated tumor tissue (*F* = 7.567, DF = 5, by ANOVA with Sidak’s posttest; *p* < 0.0001; Fig. 4d). Specifically, a significantly lower percentage of CD4+FoxP3+ T cells were seen following treatment with P/a-CTLA-4 (*p* < 0.009), P/a-PD-1 (*p* < 0.003) and P/a-CTLA-4+P/a-PD-1 (*p* = 0.0004) compared to PBS (Fig. 4d). These results were almost exactly inverse to CD8+ T cell data. A significant reduction of Tregs was also observed when comparing the a-CTLA-4 with the P/a-CTLA-4 (*p* < 0.0001) and P/a-CTLA-4+P/a-PD-1 (*p* < 0.0001) treatments (Fig. 4d). These data suggested that combination treatment was the most effective at inhibiting CD4+FoxP3+Tregs in the tumor tissue. The differences in Treg abundance between flow cytometry and immunohistochemistry could be due to tissue harvest for the latter analysis at the end of animals’ life when NIC treatment led to marked reduction in Ki67+ proliferating tumor cells and the formation of multiple tumor necroses (Supplementary Fig. 5). It is possible that Tregs were affected by developing hypoxia/necrosis that caused their reduction after NIC treatment. Overall, flow cytometry and histology results confirmed that NIC treatment stimulated local brain immune system allowing CTLs to attack the tumor (summarized in Supplementary Movie 1).

**Fig. 5:**
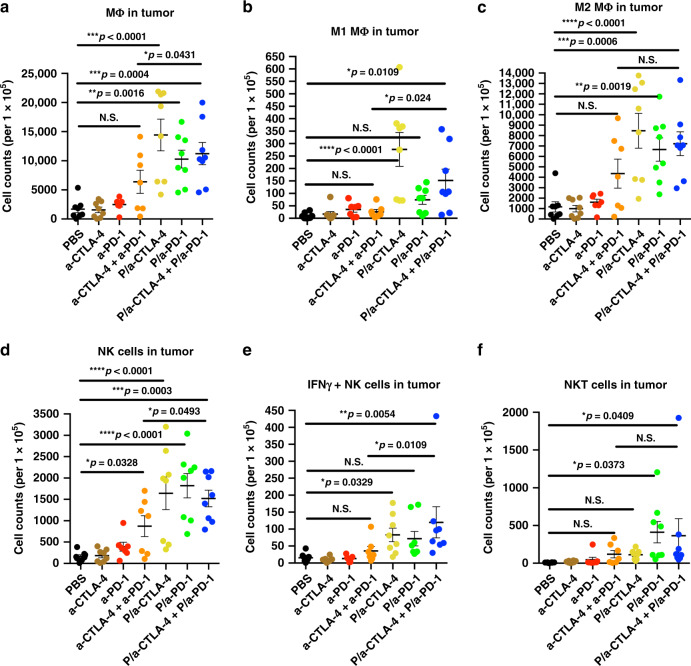
Increase of tumor macrophages, NK and NKT cells by flow cytometry. GL261 brain tumors were analyzed by the spectral flow cytometry (SONY Biotechnology) and various types of tumor-associated immune cells are presented as the cell counts in each treatment group. **a** Macrophages (MΦ): CD3-F4/80+; **b** M1 type MΦ: CD3-F4/80+iNOS+CD206-; **c** M2 type MΦ: CD3-F4/80+CD206+iNOS−; **d** Natural killer (NK) cells: CD3-NK1.1+; **e** Interferonγ+ NK cells; **f** NKT cells: CD3+NK1.1+. Approximately 100,000 events/sample are recorded and analyzed by the SA3800 software (SONY Biotechnology). Data are mean ± SEM. *p < 0.05;. **p < 0.01; ***p < 0.001; ****p < 0.0001; N.S. = non-significant (one-way ANOVA with Sidak’s posttest). *N* = 7 per group

Delivery of checkpoint inhibitors as part of NICs and activation of brain immunity resulted in significant increase of tumor macrophages (MΦ). Both total and in particular, M1 MΦ increased that are responsible for tumoricidal effect by production of iNOS to promote anti-tumor activity. Their numbers by flow cytometry were significantly higher in all NIC groups vs. PBS or free mAbs (*F* = 10.51, DF = 6, *p* = 0.0001; *F* = 8.745, DF = 6, *p* < 0.0001, respectively, by ANOVA with Sidak’s posttest; Fig. 5a, b). M2 MΦ numbers were also higher in NIC-treated groups vs. PBS (*F* = 9.125, DF = 6, by ANOVA with Sidak’s posttest, *p* < 0.0001; Fig. 5c), possibly triggered by massive hypoxic/necrotic areas in the tumor (Supplementary Fig. 5) as a result of treatment. However, the differences between NICs and free antibodies were non-significant. NK cells^10,33^ known as “tumor killers” were also increased in all NIC groups vs. PBS or free antibodies (*F* = 10.53, DF = 6, by ANOVA with Sidak’s posttest, *p* < 0.0001; Fig. 5d), as well as interferon γ producing anti-tumor IFNγ+ NK and to a lesser extent, NKT cells^34^ (*F* = 3.766, DF = 6, *p* = 0.0038; *F* = 2.447, DF = 6, *p* = 0.0385, respectively, by ANOVA with Sidak’s posttest; Fig. 5d–f).

**Fig. 6:**
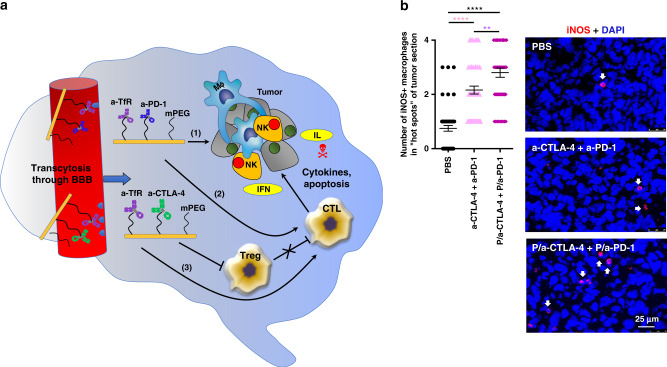
Proposed mechanism of local brain immune activation by NIC treatment. **a** The proposed mechanism of synergistic treatment with a-CTLA-4 and a-PD-1 mAbs when they cross BBB as part of a nano immunodrug. (1) a-PD-1 initiates central pathway inhibition with cancer cell attack; (2) a-PD-1 activates cytotoxic T lymphocytes; and (3) a-CTLA-4 provides local inhibition of Treg. In addition, activated macrophages (MΦ) and NK cells also contribute to tumor cell elimination. IL interleukin; IFN interferon. **b** Ex vivo study of M1 MΦ by immunostaining for iNOS (arrows) demonstrates their increased numbers in the brain tumors after combined NIC treatment as compared to PBS or a combination of free antibodies. Data are mean ± SEM. **p < 0.01; ****p < 0.0001 (ANOVA with Sidak’s posttest)

Immunohistochemical analysis for anti-tumor M1 MΦ marker iNOS was also performed on tumor sections after various treatments (Fig. 6b). The staining was nonuniform and showed evidence of “hot spots”, which were analyzed quantitatively. In accordance with flow cytometry data, immunostaining also showed significant increase of M1 MΦ after combined NIC treatment as compared with combination of free antibodies (*F* = 53.98, DF = 2, by ANOVA with Sidak’s posttest, *p* < 0.01) or PBS (*p* < 0.0001).

### NICs increase survival of GL261 glioblastoma-bearing mice

Most preclinical studies with checkpoint inhibitors used intraperitoneal (I.P.) administration^35,36^, to avoid anaphylactic shock after I.V. injection. However, systemic I.V. drug administration is widely considered as the clinically accepted method in brain cancer treatment. In animal models, we observed a rapid and fatal hypersensitivity reaction with repeated I.V. injections of a-CTLA-4, a-PD-1, and NICs during our initial experiments. Therefore, to avoid acute immune-mediated anaphylaxis-like side effects, premedication must be used^37–39^.

Although the first two I.V. treatments did not cause noticeable side effects, mice experienced a severe drop in body temperature, piloerection, loss of spontaneous activity, dyspnea, and lethargy 15–20 min after the following injections. About 20% of mice died after the third treatment and up to 100% after the fifth treatment. Different regimens to reduce treatment dosage and frequency, however, did not eliminate toxicity. To counteract these side effects, 10 mg/kg anti-histamine Triprolidine and/or 5 mg/kg platelet activating factor (PAF) antagonist CV6209 were administered prior to each drug injection following the first one. Although both Triprolidine and CV6209 reduced side effects to some level, they prevented side effects in 100% of cases only when administered as a combination regimen. Premedication allowed five repeated I.V. treatments using a 10 mg/kg antibody dose, fully comparable to the current clinical dosage of checkpoint inhibitor mAbs.

**Fig. 7:**
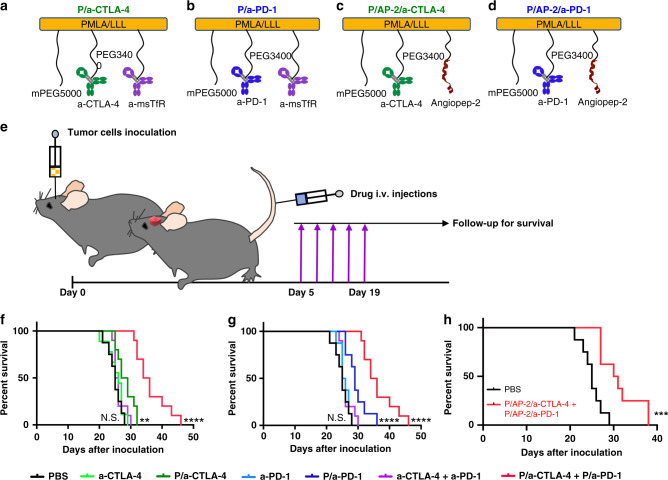
Increased GL-261-bearing animal survival after treatment with NICs. **a**–**d** Structures of treatment nanoconjugates containing PMLA/LLL as a backbone, 2% mPEG5000, a-msTfR (or AP-2), and a-CTLA-4 or a-PD-1. **e** Schematic depicting experiment workflow: tumor cells are inoculated intracranially; five I.V. drug treatments are started 5 days after inoculation after which animals (*N* = 10 per group) are followed for survival. Kaplan–Meier plot of animal survival after treatment with **f**, PBS, a-CTLA-4, P/a-CTLA-4, a-CTLA-4+a-PD-1, and P/a-CTLA-4+P/a-PD-1. Treatment with P/a-CTLA-4 and P/a-CTLA-4+P/a-PD-1 significantly increases survival compared with free antibodies, their combination and PBS. **g** Treatment with PBS, a-PD-1, a-CTLA-4+a-PD-1, P/a-PD-1, and P/a-CTLA-4+P/a-PD-1. Both NIC treatments significantly improve survival compared to free antibodies, their combination and PBS. **h** Treatment with PBS, and P/a-CTLA-4+P/a-PD-1. This experiment was performed with AP-2 peptide to cross BBB as an alternative to a-TfR antibody. NIC treatment significantly improves survival compared to PBS. Data are mean ± SEM, *N* = 6 per group. *p*-Values were obtained using a log-rank test where **p < 0.01; ****p < 0.0001; N.S. = non-significant

Survival of mice bearing intracranial GBM GL261 and treated with free mAbs or NICs alone or in combination was investigated. Both a-CTLA-4 and a-PD-1 free mAbs and their combination failed to increase survival compared to PBS treatment (Fig. 7f, g), in line with their inability to cross the BBB (Fig. 2) and increase mouse survival, as well as with the failure of a clinical trial with such mAbs as GBM treatment^9^. Crucially, either P/a-CTLA-4 or P/a-PD-1 (Fig. 7a, b) significantly improved animal survival compared to PBS (*p* < 0.008 and *p* < 0.002, respectively, by log-rank test) or the respective free antibody treatment (*p* *<* 0.04 and *p* < 0.004, respectively) (Fig. 7f, g). A combination of P/a-CTLA-4+P/a-PD-1 further improved survival (by 40% median) in GL261 tumor-bearing mice compared to PBS (*p* < 0.0001), a-CTLA-4 (*p* < 0.0001), a-PD-1 (*p* < 0.0001), P/a-CTLA-4 (*p* < 0.0001), and P/a-PD-1 (*p* < 0.004) (Fig. 7f, g). When checkpoint inhibitor mAb was attached to NIC with tumor vasculature targeting AP-2 peptide, mouse survival was also significantly improved, similar to a-TfR-mediated BBB delivery (*p* = 0.003, Fig. 7h).

### NICs increase systemic immune response

When activated, CD4+ and CD8+ T cells produce inflammatory cytokines. To examine whether NIC treatment elicited systemic immune activation, serum cytokine levels were measured by multiplex assay. Treatment with single NICs showed a slight increase of serum levels for multiple interleukins vs. PBS (Supplementary Fig. 6). However, NIC combination produced significant increases of most cytokines vs. PBS treatment (Supplementary Fig. 6A-I): IL-1β (*p* < 0.01), IL-2 (*p* < 0.05), IL-4 (*p* < 0.05), IL-5 (*p* < 0.05), IL-6 (*p* < 0.01), IL-10 (*p* < 0.05), IL-12(p70) (*p* < 0.05), and TNFα (*p* < 0.001). IFNγ also showed an increase but it was non-significant. These data suggest that the systemic immune response was also activated by NIC treatment, especially by NIC combination.

## Discussion

Cancer immunotherapy with checkpoint inhibitor mAbs represents a major advance in cancer treatment in the last decade^9^ and was recently recognized by a Nobel prize. A number of “hot” immunogenic tumors, such as melanoma and other cancers with high rate of lymphocyte infiltration, have been successfully treated with immunotherapy by activating general anti-tumor immune response. However, many brain tumor clinical trials yielded mediocre results^9^ partly because gliomas are considered “cold” and poorly infiltrated with lymphocytes^9,40^. The most significant obstacles in the treatment with checkpoint inhibitors are tumor resistance and toxicity^41^, and in the case of brain tumors, their inability to cross biological barriers^16,17^. GBM, a very aggressive tumor with short survival and with limited and poorly effective treatment options, presents a particular challenge for drug delivery because of its location in the CNS and the necessity for the drugs to cross the BBB^7^. A recent study has described survival improvement and immune system activation in glioma-bearing mice treated with free checkpoint inhibitor mAbs (a-PD1 and a-CTLA-4)^42^. Although such antibodies do not bind known receptors capable of BBB transcytosis and are unlikely to function effectively in the brain, they may still provide some activation of the brain immune system, possibly working through systemic immune stimulation^17,42^.

The glioma immune microenvironment is very complex, but, with proper checkpoint inhibitor delivery through the BBB, the basic concept for immune treatment still holds true: disrupting the PD-1/PD-L1 and CTLA-4/B7-1 complex formation in the tumor is key to the improved survival of glioma-bearing mice. We thus hypothesized that effective treatment of brain cancer with immunotherapy should involve the activation of brain local immune system by drugs able to reach the brain tumor by crossing the BBB, which cannot be readily achieved by free checkpoint inhibitor antibodies including a-CTLA-4 and a-PD-1 with clinically relevant I.V. administration. To this end, we developed polymeric NICs with covalently attached checkpoint inhibitor mAbs a-CTLA-4 and a-PD-1. They were able to cross the BBB (Fig. 2) via the proven transcytosis delivery system using polymer-conjugated a-msTfR^28,29^, or AP-2 peptide^27^, as a vehicle for nanodrugs to cross the BBB^27,43,44^ and elicit anti-tumor immune responses in brain glioma in a mouse model. NICs were immunochemically verified for their biological ability to bind their substrates, mimicking the natural conditions for naked a-CTLA-4 and a-PD-1. The satisfactory analytical results allowed us to move to the systemic drug delivery and glioma therapy.

We demonstrated the efficacy of NICs carrying a-PD-1 and a-CTLA-4 in treating GBM in a murine model vs. free a-CTLA-4 and a-PD-1. We established that NICs, as single nano agent therapies and as a combination of P/a-PD-1+P/a-CTLA-4, crossed the BBB and significantly increased survival of GL261 tumor-bearing mice activating the brain resident immune system compared to treatment with free antibodies (Supplementary Animation Video 1). To look into mechanisms behind increased animal survival, various subsets of immune cells were examined in the treated tumors. After NIC treatment compared to free mAbs, both flow cytometry and immunohistochemistry showed an increase in effector T cells (CD4+ and CD8+) that can mediate tumor attack (Figs. 3 and 4). Additionally, NK cells, another effector cell population, were increased after NIC treatment (Fig. 5). These data agreed well with increased animal survival after NIC treatment, especially with drug combination.

We also found an increase of proinflammatory M1 MΦ that are active against tumors^45^ (Figs. 5 and 6). At the same time, M2 MΦ were not reduced (Fig. 5). It has been shown that in hypoxic/necrotic tumor areas MΦ become polarized towards the M2 phenotype^45^. This could explain a persistence of M2 MΦ after NIC treatment, as pronounced tissue necrosis was found in treated tumors. This necrosis could also explain lack of tumor size reduction on MRI images after NIC treatment (Supplementary Fig. 5). GL261 tumor apparently showed typical pseudoprogression^46^ rather than real progression as was also evidenced by a marked decrease in proliferating Ki67+ cells in tumors treated with NIC but not with free antibodies (Supplementary Fig. 5).

The situation with Tregs appears to be more complicated. Immunostaining for CD4+FoxP3+ Tregs clearly showed their decrease after NIC treatment vs. free mAbs, especially after NIC combination (Fig. 4b). However, flow cytometry revealed a small Treg increase after treatment (combined NIC actually showed the smallest increase). Immunohistochemical data may be more accurate than flow cytometry quantitation because we can assess more precisely the tumor area (nearly 100%), whereas flow results would comprise tumor and adjacent non-cancerous area even with accurate tumor harvesting and only detect fully viable cells. Additionally, NIC treatment led to the formation of multiple tumor necroses, more evident at the end of animals’ life when tissue was harvested for immunostaining (Supplementary Fig. 5). Marked hypoxia/necrosis could contribute to Treg reduction after NIC treatment, similar to severe drop in Ki67+ proliferating tumor cells.

As a consequence of immune system activation, serum levels of cytokines produced by T cells to regulate immune response^47^ including IL-1β, IL-2, IL-4, IL-5, IL-6, IL-10, IL-12, and TNFα were significantly increased after combination treatment with P/a-CTLA-4+P/a-PD-1 compared to other NICs or PBS. In general, IL-1β, IL-2, IL-12, IFNγ, and TNFα are part of T helper 1 (Th1) response, whereas IL-4, IL-5, IL-6, and IL-10 are part of T helper 2 (Th2) response. IL-2 secretion promotes T and B lymphocyte activity, enhances anti-tumor immunity, stimulates microglia, and regulates Tregs^48–50^. IFNγ is mainly produced by CD4+ and CD8+ T cells, NK cells, and microglia to boost the cytotoxic immune response^50,51^. In a self-stimulating loop, the microglia can also secrete IL-12, activating NK and stimulating T cells^52^. IL-10 is a multifunctional immune cytokine with anti-angiogenic properties^53,54^. Finally, cytokines like IL-1β and TNFα can suppress tumors via stimulation of cell-mediated humoral immune reaction^55^. Some of these cytokines have been used in anti-cancer treatment: IL-4 for GBM^56,57^ IL-12 for breast cancer brain metastasis^58^, IL-2 for melanoma brain metastasis^59,60^, and IFNα and IL-2 for renal cell carcinoma^61^. CTLA-4 and PD-1 suppression also increased IL-4, IL-5, IL-6, and IL-10, which is a known effect of such treatments in other cancer types^62–64^. In our study, all these cytokines were elevated in serum of animals treated with NIC combination (Supplementary Fig. 6), supporting increased cytotoxic activity of the immune system.

Previous animal work mostly used intraperitoneal (I.P.) administration for multiple injections. It was tolerated by mice, but is not effective for the drug delivery, in particular for brain tumors shielded by BBB. Using previously undescribed premedication, we were able to safely deliver the same drugs in mice multiple times by I.V. route. Two pathways of anaphylaxis are known in mice: one is caused by antigen crosslinking of IgE on mast cells followed by release of histamine^65,66^, and the second one is mediated by IgG1 and basophil or neutrophil release of PAF^38^. Immunotherapy with a-CTLA-4 and a-PD-1 caused anaphylaxis-like side effects after repeated administrations. However, we observed absence of adverse effects after premedication consisting of anti-histamine, Triprolidine, and PAF antagonist, CV6209, previously shown to prevent anaphylaxis in other contexts^22^. In our study, premedication safely allowed for five repeated I.V. administrations at a therapeutic dosage comparable with the clinical settings. PAF inhibitor drugs are currently available for clinical use in cardiac rehabilitation and could be adopted in future clinical trials to mitigate adverse immune-mediated events related to checkpoint inhibitor therapy^38^.

Overall, our results show that BBB-crossing NICs stimulate the brain resident immune system, prompting the proliferation of CD8+ T-cells and triggering the release of several cytokines, increasing production of M1 MΦ, and thus orchestrating immune response against GBM. These NICs provide a useful tool for the delivery of immunotherapy and targeted therapies to brain tumors. They may be used for treatment of primary brain tumors and brain metastases, where current treatment is very inefficient^67^, due to the inability to deliver therapeutics through the BBB and activate brain privileged immune system.

Our regimen of premedication appears to alleviate adverse immune-mediated effects upon repeated I.V. injections of NICs, allowing the use of this clinically relevant route of administration in animal studies of checkpoint inhibitors.

In conclusion, we may highlight several important steps in our work. The presence of two antibodies in the active form on the same PMLA polymer was confirmed by state-of-the-art ELISA. We provided clear experimental evidence that nanoscale immunodrugs are efficient against GBM using several methods: flow cytometry, immunohistochemistry and the golden efficacy standard, animal survival. We compared our treatment with checkpoint inhibitor antibody delivery to brain tumors using not only anti-TfR Ab, but also AP-2 peptide. The anti-TfR Ab was used for the sake of prospective clinical trials and ELISA confirmation method for antibody, not peptides, and thus anti-TfR Ab was selected as a priority delivery vehicle. After five I.V. treatments with Triprolidine+CV6209 premedication, no animals died from toxicity, which plagues clinical trials. These data were obtained with our covalent NICs, which are different from nanoparticles. Here, the key innovative point is brain delivery of a-PD-1 and a-CTLA-4, which were never before used with “old” or “unknown” delivery vehicles, and their action in the brain, which may be used for other checkpoint inhibitors and treatments of not only untreatable GBM or brain metastases, but also of Alzheimer’s and other neurodegenerative diseases.

## Methods

### Reagents

Polymalic acid (PMLA) with molecular mass 50,000 Da (SE-HPLC/polystyrene sulfonate standards, polydispersity 1.2) was isolated from the culture supernatant of *Physarum polycephalum* M3CVII as previously described^26,68^. Trileucine (H-Leu-Leu-Leu-OH) was from Bachem. Mal-PEG3400-Mal and mPEG5000-NH2 were obtained from Laysan Bio. Rhodamine Red C2 maleimide was purchased from Thermo Fisher Scientific. Superdex G-75 was obtained from GE Healthcare. InVivoMAb anti-mouse PD-1 (clone j43, Isotype Armenian hamster IgG) was from BioXcell and mouse anti-mouse a-CTLA-4 IgG2b (clone 9D9) was from Bristol-Myers Squibb.

### Pull-down ELISA

NUNC MaxiSorp plates (Thermo Fisher Scientific) were coated with PD-1, CTLA-4 proteins (Acrobiosystems), or mouse TfR (500 ng/well) (recombinant protein made by California Institute of Technology) in coating buffer (Protein Detector™ HRP Microwell Kit; SeraCare) at 4 °C overnight. The plates were blocked with 4% skim milk for 1 h at room temperature and washed once. The samples (a-CTLA-4, a-PD-1, a-msTfR, and nanoconjugates P/a-CTLA-4 or P/a-PD-1) were incubated in binding buffer containing 0.5% milk for 1 h followed by washing four times. Secondary HRP-labeled antibodies (goat anti-rat from Abcam; goat anti-mouse and goat anti-hamster antibodies from SeraCare) were used for the detection of free and conjugated a-msTfR and conjugated a-CTLA-4 or a-PD-1. The conjugated a-msTfR was detected with anti-rat/HRP secondary antibody when the other antibody a-CTLA-4 or a-PD-1 was attached to its plate-adsorbed antigen, to confirm the presence of both antibodies on one polymer chain (pull-down ELISA). Pull-down ELISA was also performed for the detection of a-CTLA-4 or a-PD-1 when the other antibody a-msTfR was attached to its plate-adsorbed antigen similarly.

### Cell line

Mouse glioblastoma cell line GL261 was a gift from B. Badie’s lab (City of Hope Beckman Research Institute) and was cultured in Dulbecco’s modified Eagle medium (DMEM; ATCC) containing 10% fetal bovine serum with 1% mixture of penicillin (100 U/mL), streptomycin (100 μg/mL), and amphotericin B (0.25 μg/mL) at 37 °C with 5% CO_2_. This cell line is not in the database of ICLAC’s commonly misidentified cell lines. Cells were routinely checked for mycoplasma (a kit from Lonza) with negative results.

### Intracranial tumor model and treatment regimen

All animal experiments complied with all relevant ethical regulations for animal testing and research and were performed with approval of Cedars-Sinai Medical Center Institutional Animal Care and Use Committee (IACUC) No. 5289 valid until 3/31/2020.

Twenty thousand GL261 cells in 2 μL PBS were implanted intracranially into the right basal ganglia of immunocompetent 8 weeks old female C57BL/6J mice (The Jackson Laboratory). All treatments were started on the 6th day after tumor cell inoculation. Free antibodies and NICs were administered at a dose of ~10 mg/kg via tail vein injections, twice per week for a total of five injections. The tumor-bearing mice were randomized into different groups for various drug treatments a day before the treatment started. Because of the use of several experimental and control drugs plus standard control group, there was no possibility to perform blinded treatment study in order to not mix the groups. However, imaging of BBB permeation was performed using animal numbers only by researchers blinded to a specific treatment group.

To prevent anaphylactic-like adverse effects, starting with the second treatment, all mice (including the control group) received 200 μg anti-histamine Triprolidine (Sigma-Aldrich) and 100 μg platelet-activating factor (PAF) antagonist CV6209 (Santa Cruz Biotechnology) via intraperitoneal injection, respectively, 30 and 45 min prior to NIC injection. Six to ten mice per treatment group were used (flow cytometry and drug treatment), and mean value of cell counts as well as the standard error from each group were used for further analysis. Three mice were used for BBB permeation imaging and immunostaining experiments. Two or more independent experiments were performed for each assay. The number of samples per group was set to yield statistically significant data.

### Immunostaining for BBB permeation, immune cells, and cell proliferation (Ki67)

For drug delivery experiments, tumor-bearing mice alternatively injected with rhodamine-labeled NICs (P/a-CTLA-4 or P/a-PD-1 or their 1:1 mixture) or rhodamine-labeled free mAbs (a-CTLA-4 or a-PD-1 or their 1:1 mixture) (dose of mAb 10 mg/kg, rhodamine 0.1 mg/kg) were euthanized 4 h after injection. Both NICs and free mAb were labeled by rhodamine through direct attachment to mAb and each mAb was found to carry approximately one rhodamine per molecule. For T cells, macrophages, and Ki67 staining, tumor-bearing mice were alternatively treated with PBS, NICs, or free antibodies and euthanized 24 h after the third treatment. In all experiments, brains were embedded in OCT and sectioned using a Leica CM3050 S cryostat (Leica Biosystems). Tissue sections were air-dried at room temperature, fixed with 1% paraformaldehyde for 5 min and rinsed with PBS. Sections were blocked in 5% normal BSA and 0.1% Triton X-100 in PBS and incubated with anti-von Willebrand factor (vWF) to detect blood vessels (1:100, ab11713, Abcam) labeled with AlexaFluor 488 or anti-mouse CD8α (1:50, MCA609GA, Bio-Rad Laboratories), or anti-mouse CD4 (1:50, MCA4635GA, Bio-Rad), anti-mouse FoxP3 (1:50, ab20034, Abcam), anti-mouse Ki67 (1:250, ab16667, Abcam), or anti-mouse iNOS (1:100, 13120S, Cell Signaling). For Ki67 staining, sections were boiled in 10 mM citrate buffer pH 6.0 for 15 min for antigen retrieval prior to primary antibody incubation. As secondary antibodies (all at 1:200) we used goat anti-rat-FITC (112-095-167), goat anti-mouse-TRITC (115-025-166), goat anti-rabbit-TRITC (111025144), and donkey anti-rat-TRITC (712025153), from Jackson Immunoresearch. Sections were mounted with ProLongGold Antifade (Thermo Fisher Scientific) mounting medium containing 4′,6-diamidino-2-phenylindole (DAPI) to counterstain cell nuclei. Images were captured using a Leica DM6000 B microscope (Leica). Blood vessels were also detected using lectins. DyLight 488-labeled tomato lectin (DL-1174; Vector Laboratories) at 0.6 μg/μL and fluorescein-labeled RCA120 lectin (FL-1081; Vector Laboratories) at 2 μg/μL were injected as a 120 μL bolus (60 μL tomato lectin and 60 μL RCA120), 15 min prior to euthanasia.

Optical imaging data were analyzed using ImageJ Fiji software^32^. Images acquired at ×40 or ×63 magnification were first normalized for size. This was done by cropping the ×40 images (85% of data) to a dimension that equaled the ×63 image. The position of the crop was randomized so that cells included in the final analysis were objectively selected. Total cell numbers were calculated from manual counts of DAPI-labeled nuclei; counts were performed with the Fiji cell counter tool.

Cells that were labeled with anti-CD8, anti-CD4, and anti-FoxP3 antibodies were counted separately by two investigators, and ratios were calculated against the number of DAPI-labeled cells. We analyzed 3–5 images from four mice for each drug condition. In total, 74 images were analyzed to produce CD8+ data, and 79 images were analyzed to produce FoxP3+ cell counts. Statistical analysis and plots were made using Prism 7 (GraphPad). Cell counts were compared by one-way ANOVA combined with pairwise post-hoc comparisons. Quantitative analyses demonstrated significant amount of labeled NICs in all nanoconjugate fluorescence measurements in brain tumors and presented as relative fluorescence intensities (Fig. 2, Supplementary Fig. 3; a total of four mice).

### Spectral flow cytometry analysis

Mouse intracranial tumors were harvested and dissociated with 1.5 U/mL Liberase TL (Roche Diagnostics GmbH) in RPMI medium at 37 °C for 30 min. The dissociated GL261 tumor cells were filtered through 70 µm cell strainers and loaded into a 96-well plate (~10^6^/well) for flow cytometry staining. After blocking with rat anti-mouse CD16/32 (Mouse BD Fc Block, clone 2.4G2, BD Biosciences), the cells from each tumor were split into two 96-well plates and stained with a panel of T-cell antibodies and a panel of NK/macrophage antibodies in parallel (Supplementary Table 2). Flow cytometry was performed using the spectral flow cytometer SONY SA3800 (SONY Biotechnology). From each well, 100,000 events were recorded and analyzed with the SA3800 Software (SONY Biotechnology). The gating strategy for each specific population of immune cells is shown in Supplementary Figs. 3 and 4.

### Cytokine analysis

Blood was drawn from tumor-bearing mice injected with NICs 24 h after the third treatment, and serum was separated for cytokine analysis. The multiplex cytokine assay was performed using a custom Bio-Plex Pro Assay kit (Bio-Rad) including antibodies targeting IL-1β, IL-2, IL-4, IL-5, IL-6, IL-10, IL-12(p70), IFNγ, and TNFα, according to the manufacturer’s instructions. Results were obtained using a Bio-Plex 200 System (Bio-Rad) equipped with Bio-Plex Manager Software (Bio-Rad) and data were processed using the same software.

### Tumor volume measurement by MRI

To determine the tumor volume, mice were anesthetized by inhalation of isoflurane (3.5% to effect) inside an induction chamber. MultiHance, a contrast agent, was used for tumor demarcation and was dissolved in PBS at a dose of 0.4 mmol/kg, and administered via the tail vein using a 1 mL syringe and a 30-gauge needle. Anesthesia was maintained during measurements by nose cone administration of 1–1.5% isoflurane. The mouse bed was heated to prevent animal cooling during anesthesia. Breathing was regulated in the range of 45–65 breaths/min during the measurements by slightly increasing or decreasing the percentage of isoflurane. Images were recorded immediately after contrast agent injection. Spin-echo images of the entire brain were acquired. Axial slices were positioned to cover the whole brain. A multislice multiecho sequence (8 spin_echos) was used with a TR = 450 ms. Slices were acquired with a 1.0 mm thickness for a 1.8–1.8 cm field of view with a 256–196 matrix size providing an in-plane resolution of 70–92 μm/pixel. Total scan time was around 6 min. Animals underwent MRI scanning on a 9.4 T small-animal scanner (BioSpec 94/20USR, Bruker Biospin MRI GmbH). Each animal was placed inside a transmission whole body coil (T10325 V3, Bruker Biospin) with a four-channel surface array coil (T11071 V3, Bruker Biospin) positioned over the brain. The transmission body coil was used for all radio frequency transmission; the surface coil was used for detection. Representative images of MRI brain scan for a mouse from each cohort (*n* = 5) are shown (Supplementary Fig. 5). A region of interest (ROI) was drawn along the perimeter of tumors (marked by pink dotted line) and the total volume was determined for each mouse by combining all ROIs of induvial slices.

### Statistical analysis

Statistical analysis of survival data was carried out using Kaplan–Meier curves and log-rank test by GraphPad Prism 7 software. All data are presented as mean ± standard error. One-way ANOVA with Sidak’s posttest was used in Prism 7 for multiple treatment group pairwise comparisons. *P* < 0.05 was considered statistically significant.

### Reporting summary

Further information on research design is available in the Nature Research Reporting Summary linked to this article.

## Supplementary information

Supplementary Information

Description of Additional Supplementary Files

Supplementary Movie 1

Reporting Summary

## Data Availability

The original flow cytometry, drug distribution morphometry, and survival data that support the findings of this study are available from the corresponding author upon reasonable request. The authors declare that other data supporting the findings of this study including details of the nanodrug synthesis and characterization are available within the paper and its supplementary information file. The manuscript has been deposited to BioRxiv: 10.1101/466508. A reporting summary for this article is available as a Supplementary Information file.
