# Author Correction: Blood–brain barrier permeable nano immunoconjugates induce local immune responses for glioma therapy

**DOI:** 10.1038/s41467-020-20129-9

**Published:** 2020-11-26

**Authors:** Anna Galstyan, Janet L. Markman, Ekaterina S. Shatalova, Antonella Chiechi, Alan J. Korman, Rameshwar Patil, Dmytro Klymyshyn, Warren G. Tourtellotte, Liron L. Israel, Oliver Braubach, Vladimir A. Ljubimov, Leila A. Mashouf, Arshia Ramesh, Zachary B. Grodzinski, Manuel L. Penichet, Keith L. Black, Eggehard Holler, Tao Sun, Hui Ding, Alexander V. Ljubimov, Julia Y. Ljubimova

**Affiliations:** 1 Nanomedicine Research Center, Department of Neurosurgery, Cedars-Sinai Medical Center, 8700 Beverly Blvd, AHSP, Los Angeles, CA 90048 USA; 2 Bristol-Myers Squibb, 700 Bay Road, Redwood City, CA 94063 USA; 3 Department of Pathology and Laboratory Medicine, , Cedars-Sinai Medical Center, 8700 Beverly Blvd., ST 8719, West Hollywood, CA 90048 USA; 4 Department of Biomedical Sciences, Board of Governors Regenerative Medicine Institute, Cedars-Sinai Medical Center, 8700 Beverly Blvd, AHSP, Los Angeles, CA 90048 USA; 5 Samuel Oschin Comprehensive Cancer Center, Cedars-Sinai Medical Center, 8700 Beverly Blvd, Los Angeles, CA 90048 USA; 6 Harvard Medical School, 25 Shattuck Street, Boston, MA 02115 USA; 7 University of California, Los Angeles (UCLA), 621 Charles E Young Dr S, Los Angeles, CA 90095 USA; 8 Division of Surgical Oncology, Department of Surgery, David Geffen School of Medicine at University of California, Los Angeles (UCLA), 10833 Le Conte Ave, Los Angeles, CA 90095 USA; 9 Department of Microbiology, Immunology and Molecular Genetics, David Geffen School of Medicine at University of California, Los Angeles (UCLA), Los Angeles, CA USA; 10 Jonsson Comprehensive Cancer Center, University of California, Los Angeles (UCLA), 10833 Le Conte Ave, Los Angeles, CA 90095 USA; 11 The Molecular Biology Institute, University of California, Los Angeles (UCLA), 611 Charles E Young Dr E, Los Angeles, CA 90095 USA; 12 AIDS Institute, University of California, Los Angeles (UCLA), 10940 Wilshire Blvd Suite 960, Los Angeles, CA 90024 USA; 13 The California NanoSystems Institute, University of California, Los Angeles (UCLA), 570 Westwood Plaza Building 114, Los Angeles, CA 90095 USA; 14 Institut für Biophysik und Physikalische Biochemie, Universität Regensburg, Regensburg, D-93040 Germany

**Keywords:** Cancer microenvironment, CNS cancer

Correction to: *Nature Communications* 10.1038/s41467-019-11719-3, published online 28 August 2019.

The original version of this Article contained an error in Fig. 7h. The mice labelled as treated with a P/AP-2/a-PD-1+P/AP-2/a-CTLA-4 construct were inadvertently treated with a P/AP-2/a-CTLA-4+P/a-PD-1/AON c-Myc construct. The experiment has now been repeated with the correct P/AP-2/a-CTLA-4+P/AP-2/a-PD-1 construct.

In addition, in the original version of Fig. 7f and g, some mice were excluded from survival analysis due to extracranial tumor growth or possible neurogenic shock. The mice that were excluded are now shown in the Supplementary Data associated with this correction.

These errors have been corrected in both the PDF and HTML versions of the Article.

## Supplementary information

Supplementary Data 1

