# Supplementary material for: Blood–brain barrier permeable nano immunoconjugates induce local immune responses for glioma therapy
Source: Nat Commun. 2019 Aug 28;10:3850. doi: 10.1038/s41467-019-11719-3 (PMC6713723; doi:10.1038/s41467-019-11719-3)
Supplement: Supplementary file 1 — Supplementary Information [file 41467_2019_11719_MOESM1_ESM.docx]

### Supplementary Methods

**Synthesis of NICs**

**Synthesis of P/mPEG5000(2%)/LLL(40%)/2-mercapto-ethylamine (pre-conjugate)**

Pre-conjugate P/mPEG5000(2%)/LLL(40%)/2-mercapto-ethylamine was synthesized based on the method previously described1-3. At first, the pendant polymer carboxylates of PMLA (40 mg, 0.34 mmol) in 300 µL were converted into highly reactive NHS ester from the addition of N-hydroxysuccinimide (NHS) (40 mg, 0.34 mmol), dicyclohexylcarbodiimide (DCC) (74 mg, 0.34 mmol) (1:1:1 molar ratio for PMLA- COOH:NHS:DCC) in 300 µL DMF (20oC, 2 h). The ester was then substituted by amidations following the addition of mPEG5000-NH2 (34 mg, 0.7 mmol, 2% equivalent to the total malyl groups in 200 µL DMF; 1 h), H-Leu-Leu-Leu-OH (LLL) (49 mg, 0.137 mmol, 40% dissolved in 200 µL DMF with 1.1 equivalent of trifluoroacetic acid to LLL for solubilization, added in 6 portions at 10-min intervals, each followed by aliquots of 1.1 equivalent of trimethylamine to LLL), 2-mercaptoethylamine (MEA) (3.9 mg, 0.034 mmol, 10%, DMF, 30 min, together with DTT 2 equivalent to MEA, followed by 1 equivalent triethylamine). The completion of each reaction by exhausting the amine reactant was verified by ninhydrin test. After hydrolysis of unreacted NHS ester by the addition of phosphate buffer (100 mM, pH 6.8), precipitation of dicyclohexylurea and removal by filtration, the pre-conjugate P/mPEG5000(2%)/LLL(40%)/MEA(10%) was purified over PD-10 column (GE Healthcare) and lyophilized (Fig. 1a).

**Synthesis of intermediate maleimides, a-CTLA-4-Rh, and a-PD-1-Rh**

Anti-mouse TfR mAb (a-msTfR; 10 mg, 5mg/mL), anti-mouse CTLA-4 mAb (a-CTLA-4;

10 mg, 5 mg/mL) and anti-mouse PD-1 mAb (a-PD-1; 10 mg, 5 mg/mL) each was reduced with Tris (2-carboxy ethyl) phosphine hydrochloride (TCEP) (5 mM, 30 min, 20oC) in PBS. After removal of unreacted TCEP on PD-10 column (GE Healthcare), the reduced antibody was conjugated with Mal-PEG3400-Mal (10 mmole, EDTA 5 mM, 6 mL PBS, 30 min). Successful conjugation was confirmed by size exclusion HPLC (SE- HPLC) as indicated by a small consistent shift of ~0.1 min to the left of original mAb.

The maleimide derivatives of antibodies were concentrated by centrifugal filtration in Vivaspin 20 (cutoff 30 kD, Sartorius Stedim Biotech), and the product was purified over Sephadex G75 with 100 mM pH 6.3 phosphate buffer as eluent. a-CTLA-4-Rh- PEG3400-Maleimide or a-PD-1-Rh-PEG3400-maleimide were labeled with rhodamine- maleimide using similar synthesis. a-CTLA-4 or a-PD-1 (5 mg, 5 mg/mL) was reduced with TCEP as above. After removal of unreacted TCEP on PD-10 column, it was reacted with rhodamine red C2 maleimide (45 μg, 2 mg/mL) for 1 min at room temperature and to the reaction mixture was immediately added Mal-PEG3400-Mal (5 mmole, EDTA 5 mM, 3 mL PBS, 30 min). The products were concentrated and purified with G-75 column as above. The synthesis of a-CTLA-4-Rh, and a-PD-1-Rh was done similarly with TCEP reduction and reaction with rhodamine red C2 maleimide followed by PD-10 column purification but without the addition of Mal-PEG3400-Mal.

**Synthesis of full NICs, P/a-CTLA-4-Rh, and P/a-PD-1-Rh**

Pre-conjugate (P/mPEG5000(2%)/LLL(40%)/2-mercapto-ethylamine 2.5 mg/mL; 11.7 mg, in 4 mL of phosphate buffer) was added dropwise to the mixture of a-CTLA-4- PEG3400-maleimide (or a-PD-1-PEG3400-maleimide) and a-msTfR-PEG3400- maleimide (10 mg each, equal to 0.2% equivalent to total malyl groups) in 4 mL 100 mM

sodium phosphate buffer (100 mM, pH 6.3). Successful conjugation (30 min, 20oC) was confirmed by SE-HPLC. After conjugation was completed, excess of pyridyl dithiopropionate (PDP) was added to block remaining sulfhydryl groups (30 min, 20oC). The volume was adjusted to 4 mL by centrifugation over Vivaspin 20 and the product was purified over PD-10 column in PBS. After removal of endotoxin using Triton X-1144, conjugates P/mPEG5000(2%)/LLL(40%)/a-CTLA-4(0.2%)/a-msTfR(0.2%) (P/a-CTLA-4) (Fig. 3a) and P/mPEG5000(2%)/LLL(40%)/a-PD-1(0.2%)/a-msTfR(0.2%) (P/a-PD-1)

(Fig. 3a) were aliquoted and stored at -20oC. P/a-CTLA-4-Rh, and P/a-PD-1-Rh were synthesized similarly using a-msTfR-PEG3400-maleimide and a-CTLA-4-Rh-PEG3400- Maleimide, or a-PD-1-Rh-PEG3400-maleimide reacting with pre-conjugate (P/mPEG5000(2%)/LLL(40%)/2-mercapto-ethylamine.

**Size-exclusion HPLC**

The synthesis of NICs were monitored with Hitachi HPLC system equipped with diode array detector (DAD) detectors (Hitachi) using size exclusion Polysep-GFC-P 4000 column (Phenomenex). The samples were monitored at 220, 260, and 280 nm with flow rate of 1 mL/min and PBS as eluent at 25oC.

**FTIR analysis**

Solutions of antibody, pre-conjugate, and final nanoconjugates (P/a-CTLA-4 or P/a-PD-

1) (1 mg/mL) in 100 µL PBS were lyophilized, and each mixed with 150 mg KBr. The mixtures were then analyzed using a Bruker ALPHA FTIR instrument fitted with a DRIFT module. An equivalent amount of PBS sample was measured and subtracted as background.

**Hydrodynamic diameter and ζ-potential measurement**

The NICs were characterized with respect to their size (hydrodynamic diameter) using a Malvern Zetasizer Nano (Malvern Instruments). The diameter that is measured in DLS (Dynamic Light Scattering) refers to the particle diffusion within a fluid and is referred to as the hydrodynamic diameter corresponding to the diameter of a sphere that has the same translational diffusion coefficient as the NICs. All calculations were carried out using the Zetasizer 7.0 software. For the NIC size and *ζ*-potential measurements, the solutions were prepared in PBS at the concentration of 1-2 mg/mL after 0.2 μm membrane filtration. All copolymer solutions were prepared immediately before analysis at 25°C. Data represent the mean value obtained from three independent measurements.

**Malic acid assay by malate dehydrogenase**

The content of PMLA in NICs was estimated by quantitative measurement of the malic acid produced after hydrolysis of NICs based on the photometric or fluorometric measurement of NADH formed by malate dehydrogenase (MDH)-catalyzed oxidation of L-malate to oxaloacetate. This enzyme is highly specific for L-malate, even in complex mixtures such as cellular extracts. Briefly, standard malic acid and sample solutions (100 μL) containing NICs were mixed with 100 μL of 12 M hydrochloric acid and incubated for 16 h at 116ºC in a 2-mL sealed glass vial. The cooled mixture was neutralized to near-neutral with 5 N NaOH equivalent to the mole of HCl. The standard malic acid and NIC hydrolysis solutions (20 μL) were mixed with 280 μL of glycine buffer (Sigma-Aldrich), 20 μL of NAD+ (Sigma-Aldrich; 26.7 mg/mL, 40 mM), and 15 μL of L- malate-dehydrogenase (EMD Millipore; diluted 100-fold from commercial stock),

allowed to stand for 30 min at 37ºC, and the absorbance was read at 340 nm wavelength. The amount of malic acid in the sample was calculated from the standard curve.

# A B

1.00

1.00

0.75 0.75

**Abs (630 nm)**

**Abs (630 nm)**

0.50 0.50

0.25 0.25

0.00

0 250 500 750 1000 1250

**Conc (ng/mL)**

0.00

0 250 500 750 1000 1250

**Conc (ng/mL)**

# C D


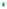

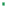

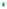

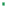

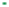

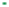

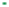

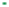

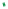

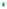

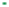

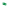

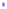

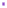

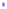

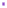

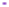

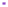

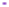

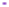

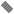

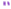

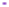

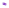

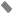

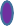

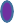

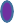

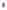

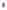

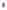

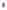

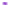

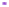

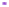

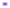

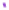

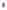

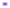

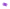

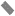

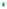

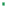

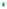

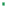

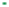

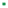

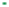

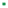

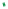

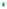

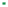

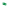

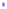

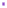

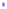

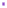

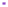

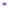

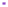

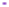

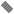

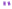

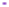

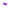

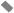

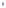

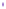

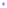

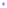

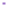

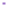

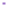

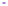

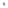

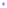

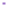

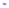

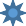

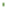

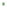

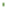

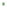

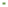

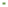

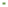

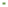

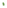

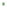

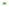

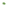

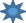

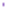

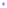

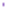

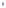

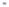

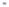

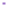

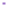


**PMLA/LLL**

**PMLA/LLL**

**PMLA/LLL**

**PMLA/LLL**

Detection of a-msTfR

Detection of a-msTfR

on conjugate

Detection of a-CTLA-4

on conjugate

Detection of a-msTfR

Detection of a-msTfR

on conjugate

Detection of a-PD-1

on conjugate

### Supplementary Figure 1. Pull-down ELISA analysis of NIC composition showing their biological activities towards surface coated mouse transferrin receptor

**A-B**, Validation of simultaneous conjugation and activity of a-msTfR and a-CTLA-4/a- PD-1 on a single platform by ELISA of P/a-CTLA-4 (**A**) and P/-a-PD-1 (**B**). **C**, Illustration of ELISA method used in **A. D**, Illustration of ELISA method used in **B**. Free a-msTfR (n); a-msTfR on conjugate (u); a-CTLA-4 (l, in **A,C**) and a-PD-1 (l, in **B,D**) on conjugate.

A

P/a-CTLA-4 a-CTLA-4

Pre-conjugate

Transmittance [%]

100 120 140 160 180 200 220 240

3500

3000

2500

2000

1500

1000

500

Wavelength cm-1

P/a-PD-1 a-PD-1

Pre-conjugate

## B

Transmittance [%]

160

180

200

3000

100

120

140

2500

2000

1500

1000

500

Wavelength cm-1

### Supplementary Figure 2. FTIR spectra of NICs

**A**, DRIFT-FTIR spectra of the pre-conjugate (khaki), the antibody a-CTLA-4 (green), and the final nanoconjugate P/a-CTLA-4 (pink). **B**, DRIFT-FTIR spectra of the pre- conjugate (green), the antibody a-PD-1 (pink), and the final nanoconjugate P/a-PD-1 (red).

### Supplementary Figure 3. Gating strategy for T cells analysis

Dissociated brain tumor cells were analyzed by flow cytometry to examine the tumor- associated T lymphocytes. 100 000 events were collected for each sample in the analysis. Cells of interest were chosen based on size in the FSC/SSC plots, and then dead cells (Ghost Dye Violet 450 positive) were excluded from analysis. Total T cell number was determined by CD3+ cell counts, and CD4+ and CD8+ T cells were further gated within CD3+ T lymphocytes.

### Supplementary Figure 4. Gating strategy for macrophage, NK and NKT cell analysis

Dissociated brain tumor cells were analyzed by flow cytometry to examine the tumor- associated macrophages (M*Φ*), NK and NKT cells. 100 000 events were collected for each sample in the analysis. Cells of interest were chosen based on size in the FSC/SSC plots, and then dead cells (Ghost Dye Violet 450 positive) were excluded from analysis. Immune cells of interest were gated as follows: M*Φ* were defined as CD3-F4/80+, NK cells were defined as CD3-NK1.1+, and the NKT cells were CD3+NK1.1+.

**H&E**

**PBS a-CTLA-4 + a-PD-1 P/a-CTLA-4 + P/a-PD-1**

**Ki67**

**50 µm**

**50 µm**

**Supplementary Figure 5. MRI and related histological changes in GL261 tumors** Magnetic resonance imaging (MRI) was performed to assess the brain tumor volume in tumor-bearing mice. Spin-echo MRI images of the entire brain were acquired from five mice/group, and representative images from PBS, a-CTLA-4 + a-PD11 and P/a-CTLA-4

+ P/a-PD-1 are shown. No statistical difference in tumor volume was observed among treatment groups. H&E staining revealed a markedly different pattern among treatment groups. Large necrotic areas were observed in NIC-treated animals compared to untreated and treated with free antibodies. Strong Ki67 staining was observed in untreated (PBS) group of animals. Animals treated with combination of free antibodies (a-CTLA-4 + a-PD-1) showed substantial Ki67 staining, but very weak and sparse staining was observed for nanoconjugate-treated group (P/a-CTLA-4 + P/a-PD-1).

**A B C**

***p*<0.01

**p*<0.05

***p*<0.01

**4000**

**p*<0.05

**p*<0.05

**p*<0.05

**p*<0.05

**p*<0.05

**p*<0.05

**3000**

**IL-1**β **(pg/ml)**

**2000**

**1000**

**0**

**PBS P/a-CTLA-4 P/a-PD-1 P/a-CTLA-4 + P/a-PD-1**

**1400**

**1300**

**1200**

**1100**

**1000**

**IL-2 (pg/ml)**

**900**

**800**

**700**

**600**

**500**

**400**

**300**

**200**

**100**

**0**

**PBS P/a-CTLA-4 P/a-PD-1 P/a-CTLA-4 + P/a-PD-1**

**850**

**800**

**750**

**700**

**650**

**600**

**IL-4 (pg/ml)**

**550**

**500**

**450**

**400**

**350**

**300**

**250**

**200**

**150**

**100**

**50**

**0**

**PBS P/a-CTLA-4 P/a-PD-1 P/a-CTLA-4 + P/a-PD-1**

**D E F**

**1250**

**p*<0.05

**p*<0.05

**p*<0.05

***p*<0.01

****p*<0.001

***p*<0.01

**1000**

**750**

**IL-5 (pg/ml)**

**500**

**500**

**400**

**300**

**IL-6 (pg/ml)**

**200**

**800**

**700**

**p*<0.05

**p*<0.05

**p*<0.05

**600**

**IL-10 (pg/ml)**

**500**

**400**

**300**

**250**

**0**

**G 3000**

**p*<0.05

**p*<0.05

**2500**

**IL-12(p70) (pg/ml)**

**2000**

**PBS P/a-CTLA-4 P/a-PD-1 P/a-CTLA-4 + P/a-PD-1**

**100**

**0**

**H 4000**

**3000**

**TNF**α **(pg/ml)**

**PBS P/a-CTLA-4 P/a-PD-1 P/a-CTLA-4 + P/a-PD-1**

****p*<0.001

*****p*<0.0001

****p*<0.001

**200**

**100**

**0**

**I 400**

**300**

**PBS P/a-CTLA-4 P/a-PD-1 P/a-CTLA-4 + P/a-PD-1**

**1500**

**2000**

**200**

**IFN**γ **(pg/ml)**

**1000**

**500**

**1000**

**100**

**0**

**PBS P/a-CTLA-4 P/a-PD-1 P/a-CTLA-4 + P/a-PD-1**

**0**

**PBS P/a-CTLA-4 P/a-PD-1 P/a-CTLA-4 + P/a-PD-1**

**0**

**PBS P/a-CTLA-4 P/a-PD-1 P/a-CTLA-4 + P/a-PD-1**

### Supplementary Figure 6. Multiplex assay for cytokine expression in blood after treatments.

Cytokine expression levels in sera from mice alternatively treated with PBS, P/a-CTLA- 4, P/a-PD-1, and P/a-PD-1 + P/a-CTLA-4. **A,** IL-1β, **B,** IL-2, **C,** IL-4, **D,** IL-5, **E,** IL-6, **F,**

IL-10, **G,** IL-12(p70), **H,** TNF*α***,** and **I,** IFN*γ*. Combination therapy produced a statistically significant increase *vs.* PBS or single NICs in measured serum cytokines except for IFN*γ* (although the same trend was observed). Data are mean ± SEM. P values were obtained after pairwise comparisons using ANOVA with Sidak’s posttest. N=3-9 in each group.

### Supplementary Table 1. Dosage and characterization of NICs for *in vivo* treatment

| Parameter | PBS | a-CTLA-4 | a-PD-1 | P/a-CTLA-4 | P/a-PD-1 | P/a-CTLA-4 + P/a-PD-1 |
| --- | --- | --- | --- | --- | --- | --- |
| Number of mice/group | n=6 | n=6 | n=6 | n=8 | n=8 | n=10 |
| Size of PMLA | N/A | N/A | N/A | 50 kD | 50 kD | 50 kD |
| Dose (a-PD-1) | N/A | N/A | 10 mg/kg | N/A | 10 mg/kg | 10 mg/kg |
| Dose (a-CTLA-4) | N/A | 10 mg/kg | N/A | 10 mg/kg | N/A | 10 mg/kg |
| a-PD-1 and a-CTLA-4  per injectiona | N/A | 220 μg | 220 μg | 220 μg | 220 μg | 220 μg a-CTLA-4  220 μg a-PD-1 |
| Malic acid per injectionb | N/A | N/A | N/A | 92 μg | 74 μg | 166 μg |
| Loading of mAb | N/A | N/A | N/A | 0.21% | 0.26% | 0.24% |
| Size (nm)c | N/A | 12.9 | 12.9 | 28.2 | 28.0 | 28.5 |
| ζ-potential (mV)c | N/A | -2.8 | -2.3 | -11.0 | -9.9 | -9.9 |
| Endotoxin (EU/mL)d | N/A | <0.1 | <0.1 | <0.1 | <0.1 | <0.1 |

a The amount of a-PD-1 and a-CTLA-4 per injection was quantitated with protein CA assay and was normalized to the dose for treatment.

b The amount of malic acid was determined by malate dehydrogenase assay. Data represent the mean value of n=2 measurements.

c Size and ζ-potential of NICs were determined by Zetasizer Nano (Malvern Instruments). Data represent the mean value obtained from n=3 independent measurements.

d Endotoxin in NICs was removed use phase separation method and measured with Endotoxin Assay kit (Genscript).

### Supplementary Table 2. Antibody panel for spectral flow cytometry analyses

**T cells flow panel (SA3800)**

| Marker | CD3 | CD4 | CD8 | CD69 | IFNγ | FoxP3 | Ki67 | Viability dye |
| --- | --- | --- | --- | --- | --- | --- | --- | --- |
| Antigen location | Extracellular | Extracellular | Intracellular | Extracellular | Intracellular | Intracellular | Intracellular | Amino group |
| Fluorophore | BV421 | APC-Cy7 | BB515 | BV711 | PE | AF647 | PerCP- Cy5.5 | Ghost Dye Violet 450 |
| Manufacturer & Cat. No. | BD 564008 | Biolegend 100414 | BD 564422 | BD 740664 | BD 554412 | BD 560401 | BD 561284 | Tonbo Bioscience 13-0863-T500 |
| Ex/Em (nm) | 405/421 | 650/774 | 490/515 | 405/711 | 565/575 | 650/668 | 482/695 | 404/448 |
| Dilution | 1:40 | 1:40 | 1:40 | 1:40 | 1:40 | 1:40 | 1:40 | 1:1000 |

**NK cells and macrophages flow panel (SA3800)**

| NK & Macrophage | CD3 | NK1.1 | IFNγ | F4/80 | CD206 | iNOS | Viability dye |
| --- | --- | --- | --- | --- | --- | --- | --- |
| Antigen location | Extracellular | Extracellular | Intracellular | Extracellular | Extracellular | Intracellular | Amino group |
| Fluorophore | BV421 | BV605 | PE | BV711 | PE-Cy7 | APC | Ghost Dye Violet 450 |
| Manufacturer & Cat. No. | BD 564008 | Biolegend 108739 | BD 554412 | BD 565612 | Biolegend 141720 | eBioscience 17-5920-82 | Tonbo Bioscience 13-0863-T500 |
| Ex/Em (nm) | 405/421 | 407/602 | 565/575 | 405/711 | 496/785 | 650/660 | 404/448 |
| Dilution | 1:40 | 1:40 | 1:40 | 1:40 | 1:40 | 1:40 | 1:1000 |

Two separate panels of flow antibodies were designed for the Spectral flow cytometry assays using the Sony SA3800 flow cytometer (SONY Biotechnology). A cell viability dye Ghost Dye Violet 450 was used to exclude the dead cells from final data analysis. Ex/Em, excitation/emission.

### References:

1. Ljubimova, J.Y. *et al.* Polymalic acid-based nano biopolymers for targeting of multiple tumor markers: an opportunity for personalized medicine? *J. Vis. Exp.* **88**, e50668, (2014).
2. Patil, R. *et al.* MRI virtual biopsy and treatment of brain metastatic tumors with targeted nanobioconjugates: nanoclinic in the brain. *ACS Nano* **9,** 5594-5608, (2015).
3. Ding, H. *et al.* Inhibition of brain tumor growth by intravenous poly (*β*-L-malic acid) nanobioconjugate with pH-dependent drug release [corrected]. *Proc. Natl. Acad. Sci. U. S. A.* **107,** 18143-18148, (2010).
4. Aida, Y., Pabst, M.J. Removal of endotoxin from protein solutions by phase- separation using Triton X-114. *J. Immunol. Methods* **132,** 191-195, (1990).
