# Supplementary material for: Blood–brain barrier permeable nano immunoconjugates induce local immune responses for glioma therapy
Source: Nat Commun. 2019 Aug 28;10:3850. doi: 10.1038/s41467-019-11719-3 (PMC6713723; doi:10.1038/s41467-019-11719-3)
Supplement: Supplementary file 2 — Description of Additional Supplementary Files [file 41467_2019_11719_MOESM2_ESM.docx]

**Description of Supplementary Files**

**File Name:** **Supplementary Movie 1**

**Description:** The movie shows in a cartoon form the stages of the NIC action including BBB permeation by receptor-mediated transcytosis, Treg inactivation in the tumor microenvironment, tumor cell killing by T cells, and NIC degradation.
